# A New Editor-in-Chief Takes Over

**DOI:** 10.5334/jbr-btr.1004

**Published:** 2015-12-30

**Authors:** Jacques Pringot

**Affiliations:** 1 Emeritus Editor-in-Chief, Journal of the Belgian Society of Radiology, Belgium

## Abstract

A reformatted journal demands a new Editor-in-Chief. Over the last year, the paper-printed Journal of the Belgian Society of Radiology has gone through a radical and successful transformation into a fully electronic open medium on an advanced IT platform. The time has thus become right for me to step down as Editor-in-Chief

A reformatted journal demands a new Editor-in-Chief. Over the last year, the paper-printed Journal of the Belgian Society of Radiology has gone through a radical and successful transformation into a fully electronic open medium on an advanced IT platform. The time has thus become right for me to step down as Editor-in-Chief

Last November, during the annual Symposium of the Belgian Society of Radiology, President Geert Villeirs appointed Dr Alain Nchimi as the new Editor-in-Chief. Dr Nchimi has earned his Medical Degree and completed a Master Degree in Medical Imaging at the University of Liège. In 2015 he obtained a PhD from the same University. He acquired a diversified professional experience successively in paediatric radiology and nuclear medicine at Great Ormond Street Hospital in London and at the Centre Hospitalier Chrétien in Liège in body imaging and paediatric radiology in the Medical Imaging Department as a consultant, and finally as the Head of the Cardio-Vascular and Thoracic Imaging Department in the Medical Imaging Department of the University Hospital of Liège. He became an Associated Professor of Medical Imaging in 2015. Currently he is a Body Imaging and Paediatric Radiologist in the Medical Imaging Department of the Hospital Center in Luxembourg. He is a member of the Belgian Society of Radiology chairing the Section of Cardio-Vascular Imaging, the European Society of Radiology, the European Societies of Thoracic Imaging and of Cardiac Imaging, as well as the European Association of Cardio-Vascular Imaging and the European Society of Cardiology. Dr Nchimi has published numerous book chapters and peer-reviewed research papers in national and prominent international journals such as Radiology, AJR, British Journal of Radiology, and European Radiology. He has served as a reviewer in at least nine radiological and clinical journals among which are JBR-BTR, European Radiology, AJR, Circulation, and the The International Journal of Cardio-Vascular Imaging. Dr Alain Nchimi’s exemplary experience in clinical research and academic radiology qualifies him highly for the position he has been newly appointed for.

The Journal of the Belgian Society of Radiology (JBSR) is the follower of a more than 100-year-old national radiological journal founded in 1905. Over the years the journal has changed its title, its format, the language used, and authorship [1]. The original title in French, Journal Belge de Radiologie (J.belg.radiol), continued until 1976. Subsequently, it became bilingual (Journal Belge de Radiologie-Belgisch Tijdschrift voor Radiologie (J.belg.radiol-Belg.t.radiol)) and later the acronym JBR-BTR was used until June 2015.

In 1995 the content of the journal was restricted to diagnostic and interventional radiology, related sciences and continuing education. The journal then started publishing in English and, over the last couple of decades; the authorship grew to become more international. During its long existence the journal was constantly supported as the organ of the Belgian Society of Radiology by the board and its subscribers, mostly the members of the society. The number of non-members subscribing, often through international libraries, remained stable and contributed to the diffusion of the journal among others at the Harvard Medical School and the Chinese University of Shanghai. During the last thirty years indirect sponsoring by radiological firms increased in importance, particularly under the action of the last two editorial managers, Daniel Meire and Patrick Seynaeve.

The swift implementation of the decision of the Board to move to the electronic-only edition of the journal was more complicated than expected, the devil lying in many details. However, thanks to the outstanding efficiency of the editorial team, in particular the Associate Editor Piet Vanhoenacker and the Ubiquity Press Editorial Manager Sam Hall, as well as with the support of the president of the Board and of the Scientific Committee, publication of the JBSR on the dedicated website was only slightly delayed. Issue two is more delayed due to the backlog of manuscripts to be edited after the closure of the Brussels editorial office last September – a temporary collateral damage.

Looking ahead, the future of the JBSR is in great hands for the best development with the advanced Ubiquity Press platform.

Personally I am very proud of what has been accomplished together with the editorial team during the last 40 years. In particular Mrs Jocelyne Burion, who was in charge of the processing and editing of the manuscripts, has been of immense help.

I am confident about the bright future of the JBSR and look forward to its future development.

Jacques Pringot

Emeritus Editor-in-Chief
